# Phalangeal Bone Mineral Density Mapping Using Quantitative CT: Implications for Hand Surgery Fixation Planning

**DOI:** 10.3390/diagnostics16121843

**Published:** 2026-06-15

**Authors:** Zoe K. Papadopoulou, Konstantinos N. Malizos, Filippos Filippou, Vasileios Raoulis, Alexis T. Kermanidis, Michail E. Klontzas, Aristidis H. Zibis

**Affiliations:** 1 Department of Anatomy, Faculty of Medicine, University of Thessaly, 41500 Larissa, Greece; kmalizos@otenet.gr (K.N.M.); v_raoulis@yahoo.gr (V.R.); ahzibis@gmail.com (A.H.Z.); 2 Department of Mechanical Engineering, School of Engineering, University of Thessaly, 38334 Volos, Greeceakermanidis@mie.uth.gr (A.T.K.); 3 Artificial Intelligence and Translational Imaging Lab, Department of Radiology, School of Medicine, University of Crete, 70013 Heraklion, Greece; miklontzas@uoc.gr

**Keywords:** bone mineral density (BMD), phalangeal base, quantitative CT, BMD mapping, Hounsfield units, tendon-to-bone fixation, hand surgery

## Abstract

**Objective:** To quantify and map bone mineral density (BMD) at the bases of human finger phalanges using computed tomography (CT) with a calibration phantom and to compare BMD both between and within digits. **Methods:** Ten cadaveric hands (H1 to H10) were CT scanned with a Model 3 CT Calibration Phantom (Mindways). All data were processed in the Horos software (Version 4.0.0) and the regions of interest (ROIs) at each phalangeal base were delineated. Hounsfield Units (HU) were converted to BMD (mg/cm^3^) per the phantom framework. Descriptive statistics and repeated-measures ANOVA analyses were performed for each digit and corresponding phalangeal level (proximal, middle, distal). Inter-digital comparisons were performed at corresponding phalanx levels and intra-digital variations were analyzed within digits across phalangeal levels. **Results:** Mean BMD varied across digits and phalangeal levels. At the proximal phalanx base, the thumb and index fingers exhibited the highest values, whereas at the middle phalanx base the middle and ring fingers demonstrated the highest mean BMD values. At the distal phalanx base, the little finger demonstrated the highest BMD value, while the lowest value was observed at the distal phalanx of the index finger. Intra-digital analysis revealed distinct distribution patterns: BMD decreased distally in the thumb and index fingers, peaked at the middle phalanx in the middle and ring fingers, and was highest distally in the little finger. Repeated-measures ANOVA demonstrated statistically significant intra-digital differences in the thumb and index fingers, whereas no statistically significant inter-digital differences were observed across corresponding phalangeal levels. **Conclusions:** CT-based, phantom-calibrated BMD mapping at the bases of the phalanges demonstrates substantial intra-digital variability and descriptive inter-digital differences. These site-specific findings may provide additional information relevant to implant selection and preoperative planning for fixation in phalangeal fractures and tendon- or ligament-to-bone insertion injuries in hand surgery.

## 1. Introduction

The assessment of bone quality in the phalanges is particularly important for tendon-to-bone and ligament reinsertion procedures in hand surgery, where local bone mineral density may influence fixation strength and healing at the insertion site. This is especially relevant in tendon avulsion injuries, in which secure reattachment depends on the mechanical competence of the underlying bone. At the same time, bone quality also plays a critical role in the management of phalangeal fractures and dislocations. However, direct evaluation of phalangeal bone density and strength remains challenging due to the small size of the bones, their complex anatomy, the variability in functional loading across digits, and inter-individual factors such as age and occupational use.

Computed tomography (CT) can be used to assess bone mineral density (BMD), and CT-based approaches—including phantom calibration—enable quantitative or semi-quantitative evaluation of bone quality beyond plain radiography [1]. In parallel, Hounsfield Unit (HU)-based measurements from routine CT have been explored for opportunistic screening of low bone density, supporting the broader feasibility of CT-derived density metrics in clinical workflows [2]. Prior work has also examined phalangeal BMD in relation to skeletal strength measures, highlighting that density information at the hand may carry clinically relevant biomechanical implications [3]. Nevertheless, site-specific mapping of BMD at the bases of the finger phalanges—regions directly involved in implant anchorage and insertion-related procedures—has not been consistently characterized across digits.

From a surgical perspective, fixation stability and the success of tendon- or ligament-to-bone repair depend critically on the local bone quality at the insertion or anchorage site. In small bones such as the phalanges, even subtle variations in BMD may be relevant to screw purchase, anchor fixation, and the risk of fixation failure. Recent advances in quantitative CT-based bone assessment and opportunistic skeletal imaging have further expanded interest in regional and site-specific evaluation of bone quality in musculoskeletal surgery [4,5]. Accordingly, a more detailed understanding of phalangeal BMD distribution could provide clinically relevant information beyond global or averaged skeletal measurements and may support preoperative fixation planning and implant selection.

Therefore, the aim of the present study was to quantify phantom-calibrated BMD at the bases of the proximal, middle, and distal phalanges in cadaveric human hands and to map its distribution through inter- and intra-digital comparisons. In the context of the present study, “BMD mapping” refers to the comparative assessment of site-specific BMD distribution at the bases of the phalanges across digits and phalangeal levels, potentially providing an anatomical reference framework for preoperative fixation planning. We hypothesized that measurable inter- and intra-digital variability in BMD distribution would be present across phalangeal levels, and that identifying level- and digit-specific regions of higher density could support preoperative planning for fracture fixation and for ligament-to-bone and tendon-to-bone reinsertion procedures.

## 2. Materials and Methods

### 2.1. Specimen Preparation

**Figure 1:**
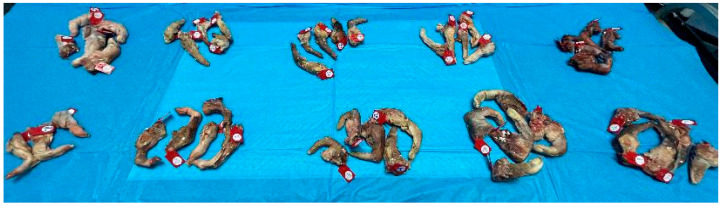
Overview of thawed and labeled cadaveric digit specimens (48 digits, 134 phalanges), grouped by hand of origin prior to CT scanning, with visible specimen identification labels.

Ten cadaveric hands (H1–H10), comprising 48 intact digits and 134 phalanges, were analyzed (specimen H6 lacked the ring and little fingers). The specimens were derived from male and female donors aged between 52 and 78 years; information regarding occupation was not available. All specimens were stored frozen and thawed to room temperature prior to imaging to minimize dehydration-related artifacts. Each hand had been previously dissected, and the digits were separated prior to scanning (Figure 1). Data on hand dominance were not available.

For each specimen, the five digits were arranged sequentially from thumb to little finger on the CT table, approximating their natural anatomical order and spacing. Because the digits had been previously frozen, they retained varying degrees of flexion or extension and could not be positioned in a completely standardized straight alignment. No attempt was made to force all joints into extension; instead, each digit was placed on the calibration phantom surface in its natural resting configuration. This approach was intended to minimize mechanical stress while preserving approximate anatomical relationships across digits.

### 2.2. CT Image Acquisition

Each group of digits was scanned individually using a 16-slice CT scanner (LightSpeed 16, GE HealthCare, Chicago, IL, USA) in conjunction with a Model 3 CT Calibration Phantom (Mindways, Austin, TX, USA). Scanning parameters were tube voltage 120 kVp, tube current 200 mA, gantry rotation time 0.5 s, pitch 1.375, field of view 50 cm, slice thickness 1.25 mm, and image matrix 512 × 512, according to manufacturer specifications [6,7]. Reconstruction was performed using a bone algorithm kernel with a 1.25 mm slice increment. All datasets were visually inspected for motion and beam-hardening artifacts; none were excluded. The estimated CTDIvol was <15 mGy per scan.

### 2.3. ROI Definition and HU Measurement

**Figure 2:**
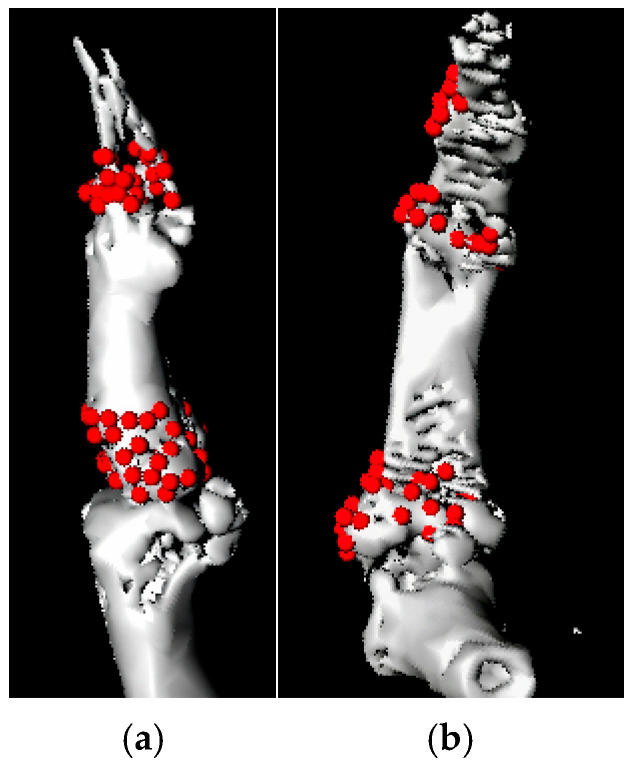
(**a**) Three-dimensional rendering of a representative digit specimen showing the delineated boundaries of each phalangeal base projected onto the surface model to minimize positional bias and guide consistent region-of-interest (ROI) placement on axial CT slices (lateral view). (**b**) The same three-dimensional rendering shown in an oblique view to better illustrate the spatial relationship of the delineated boundaries. The red markers represent the projected ROI boundary points used to delineate each phalangeal base on the three-dimensional surface model.

Image analysis was performed using HorosVersion 4.0.0 (macOS platform). For each phalanx of every digit, a region of interest (ROI) was manually defined at the base of the phalanx, corresponding to clinically relevant fixation and tendon-insertion sites in hand surgery. The superior and inferior boundaries of each phalangeal base were delineated on multiplanar reconstructions (MPRs), and Hounsfield Units (HU) were measured within each ROI. These anatomical limits were projected onto the three-dimensional reconstruction to ensure consistency and minimize positional bias from non-orthogonal sections (Figure 2a,b).

All ROI placements and measurements were performed by a single observer with experience in musculoskeletal imaging. Standardized anatomical landmarks and multiplanar reconstructions were used to ensure consistency across all measurements.

### 2.4. Data Processing and BMD Conversion

**Figure 3:**
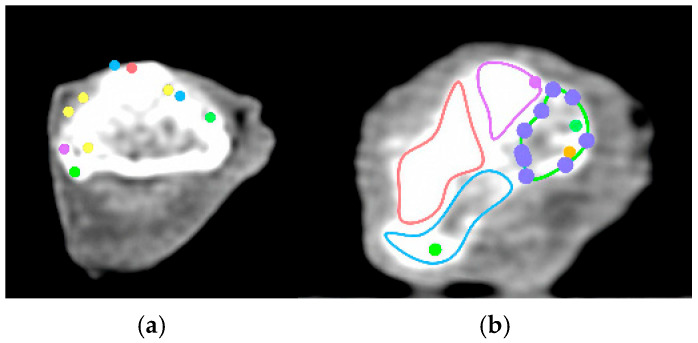
(**a**) Axial CT slice demonstrating the projection of predefined reference points identified on the 3D reconstruction. These points were used to localize anatomically consistent regions for subsequent quantitative analysis. The colored points represent the projected boundary points defining the phalangeal base boundaries. (**b**) Manual delineation of regions of interest (ROIs) on axial CT images using the closed-polygon tool. The ROIs were defined based on the previously identified reference points to ensure consistent anatomical sampling. Corresponding Hounsfield Unit (HU) measurements were obtained from each ROI. The purple points illustrate the sequential placement of points used to construct the closed polygon, while the colored closed lines represent the resulting polygonal ROI boundaries. Additional isolated colored points represent boundary reference points used during ROI delineation. Different colors are used solely for visual distinction. Different colors are used solely for visual distinction and do not represent different anatomical structures, boundary types, or measurement categories.

Based on predefined anatomical boundaries (Figure 2a,b), cross-sectional axial CT slices were selected for analysis (Figure 3a). Within each slice, regions of interest (ROIs) were manually delineated using the closed polygon tool in Horos software, and the mean Hounsfield Unit (HU) value for each ROI was recorded (Figure 3b).

The number of polygonal ROIs per phalanx ranged from 3 to 83 (mean: 14.54). All HU measurements were exported and organized into spreadsheets for further analysis. For each phalanx base, the minimum, maximum, and mean HU values, as well as the total number of ROIs, were calculated and documented.

**Figure 4:**
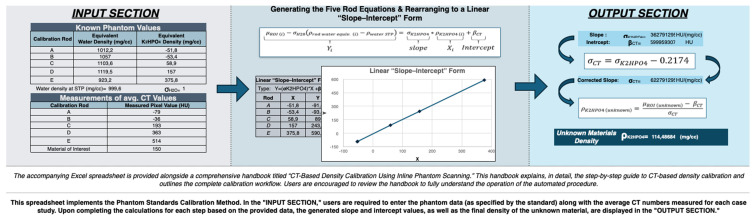
Workflow of the conversion algorithm used to transform Hounsfield Unit (HU) values into bone mineral density (BMD, mg/cc) using the Mindways Model 3 CT calibration phantom [7]. Known phantom densities and corresponding CT numbers are used to derive a linear regression (slope and intercept), which is subsequently applied to convert HU measurements of phalangeal samples into equivalent BMD values.

An algorithm was developed using the phantom reference framework and automated in Microsoft Excel to streamline data processing (Figure 4). Through this procedure, all measured HU values were converted into volumetric BMD equivalents (mg/cm^3^), using the manufacturer-provided calibration equations and reference materials. Calibration was performed using linear regression analysis between the measured CT numbers of the phantom rods and their corresponding manufacturer-provided K2HPO4-equivalent reference densities, according to the manufacturer’s protocol. The resulting slope and intercept values were incorporated into the spreadsheet-based conversion algorithm, and volumetric BMD values were calculated using the following equation:

ρK2HPO4 = (μROI − βCT)/σCT

where μROI represents the measured HU value within the ROI, βCT the calibration intercept, and σCT the calibration slope. A single standardized calibration equation was applied consistently across all scans because all specimens were acquired using the same CT acquisition protocol and phantom configuration [7]. Following conversion, the data were reclassified and organized into summary tables (Tables S1–S10), while the full dataset is provided in the Supplementary Materials.

The number of polygonal ROIs per phalanx varied across specimens due to differences in phalangeal orientation, slice geometry, and local anatomical bone availability. For each phalangeal base, a predefined three-dimensional anatomical region was analyzed across consecutive axial slices. On each slice, closed polygonal ROIs were drawn, and each ROI represented the mean HU value of all pixels within the enclosed area. The final BMD value for each phalangeal base was calculated as the average of all ROI mean values across all slices. Because each ROI already represents the mean value of all enclosed pixels, dividing the same anatomical area into multiple smaller ROIs or fewer larger ROIs ultimately yields the same overall mean value, provided that the entire predefined anatomical region is covered across the analyzed slices. Therefore, variability in the number or size of ROIs was not expected to systematically influence the final mean BMD measurements or materially affect measurement accuracy.

### 2.5. Statistical Analysis

Descriptive statistics were calculated for all phalangeal measurements and are presented as mean ± standard deviation (SD). Repeated-measures analysis of variance (ANOVA) was used to evaluate intra-digital differences in BMD across phalangeal levels (proximal, middle, distal) within each digit, as well as inter-digital differences at corresponding phalangeal levels. For the thumb, comparisons were performed between the proximal and distal phalangeal levels only. Statistical significance was defined as *p* < 0.05. Statistical analyses were performed using Jamovi software (version 2.6.45).

### 2.6. Ethics Statement

Ethical approval was not required for this study because the analyses were performed on anonymized cadaveric specimens provided through institutional body donation procedures. No identifiable donor information was available.

### 2.7. Data Availability

The HU and BMD datasets, as well as the spreadsheet-based conversion algorithm developed for this study, are available from the corresponding author upon reasonable request.

### 2.8. Generative AI Statement

Generative AI was not used to generate scientific data, figures, or analytical content in this study.

## 3. Results

### 3.1. Inter-Digital BMD Comparisons by Phalanx Level

Mean bone mineral density (BMD, mg/cm^3^) values varied across digits at each phalangeal level.

At the proximal phalanx base, the ranking by mean BMD was as follows: thumb (276.35) > index finger (270.52) > middle finger (253.64) > ring finger (249.18) > little finger (230.65).

At the middle phalanx base, the ranking was as follows: middle finger (266.41) > ring finger (263.89) > little finger (251.59) > index finger (244.80). (The thumb does not have a middle phalanx.)

At the distal phalanx base, the ranking was as follows: little finger (254.45) > ring finger (240.71) > middle finger (235.36) > thumb (212.58) > index finger (198.39).

Descriptive analysis demonstrated level-specific variability in BMD distribution among digits (Table 1). Notably, higher proximal BMD values were observed in the thumb and index finger, whereas the highest mean BMD values were identified at the middle phalanx in the middle and ring fingers and at the distal phalanx in the little finger. In contrast, the lowest mean BMD value was observed at the distal phalanx of the index finger.

No statistically significant difference in BMD was observed among the proximal phalanges of the different digits.

No statistically significant difference in BMD was observed among the middle phalanges of the different digits.

No statistically significant difference in BMD was observed among the distal phalanges of the different digits (Table 2).

### 3.2. Intra-Digital BMD Patterns

Within-digit comparisons demonstrated distinct variations in BMD distribution across phalangeal levels for each digit (Table 1)):

- Thumb: Proximal phalanx base > distal phalanx base.
- Index finger: Proximal phalanx base > middle phalanx base > distal phalanx base.
- Middle finger: Middle phalanx base > proximal phalanx base > distal phalanx base.
- Ring finger: Middle phalanx base > proximal phalanx base > distal phalanx base.
- Little finger: Distal phalanx base > middle phalanx base > proximal phalanx base.

Repeated-measures ANOVA demonstrated a statistically significant difference in BMD between the proximal and distal phalanx bases of the thumb.

Repeated-measures ANOVA also demonstrated a statistically significant difference in BMD among the proximal, middle, and distal phalanx bases of the index finger.

No statistically significant difference in BMD was observed among the proximal, middle, and distal phalanx bases of the middle finger.

No statistically significant difference in BMD was observed among the proximal, middle, and distal phalanx bases of the ring finger.

No statistically significant difference in BMD was observed among the proximal, middle, and distal phalanx bases of the little finger (Table 3).

## 4. Discussion

The present cadaveric quantitative CT analysis demonstrated substantial intra-digital variability and descriptive inter-digital differences in bone mineral density (BMD) at the bases of the phalanges within the same hand. Inter-digital comparisons showed that the highest mean BMD values were observed in the thumb and index finger at the proximal phalanx level, in the middle and ring fingers at the middle phalanx level, and in the little finger at the distal phalanx level. Within each digit, distinct intra-digital patterns were identified, with the highest mean BMD values occurring proximally in the thumb and index fingers, at the middle phalanx in the middle and ring fingers, and distally in the little finger. Repeated-measures ANOVA demonstrated statistically significant intra-digital differences in the thumb and index fingers, whereas no statistically significant differences were observed in the remaining digits or across corresponding phalangeal levels between different digits. Overall, these findings suggest level-dependent variation in local bone density distribution across the hand. This structured, site-specific mapping of BMD across phalangeal bases extends beyond previous descriptive assessments of bone density in the hand. Collectively, these findings suggest the potential utility of site-specific BMD mapping when planning fixation strategies and tendon- or ligament-to-bone reinsertion procedures in hand surgery [8,9,10].

The mechanisms underlying the observed distribution patterns were not directly examined in this study; however, the observed descriptive differences between digits are consistent with the concept that local bone quality may reflect long-term functional loading and region-specific adaptation. In this context, higher BMD at the proximal bases of the thumb and index finger may relate to the distinct mechanical demands placed on these digits during pinch and precision tasks, whereas the observed variation among the ulnar digits may correspond to their roles in power grip and grasp stabilization [8,9,10]. Although basic demographic data (age and sex) were available, future studies incorporating more comprehensive donor characteristics and functional correlates are needed to clarify the relative contributions of biological and use-related factors.

From a clinical perspective, the observed level-dependent and descriptive digit-related BMD patterns may be relevant to common injury scenarios and fixation targets. For example, avulsion injuries around the thumb metacarpophalangeal joint—such as ulnar collateral ligament avulsion (“gamekeeper’s thumb”)—involve regions that, in the present dataset, exhibited descriptively higher BMD at the proximal base, which may provide a comparatively more favorable local bone support for fixation using anchors or pull-out techniques [8,9,10,11]. Similarly, the descriptively higher mean BMD observed at the middle phalanx base of the middle and ring fingers may be pertinent to extensor tendon avulsions and proximal interphalangeal (PIP) fracture-dislocations requiring stable fixation and reattachment [12,13]. At the distal level, the higher mean BMD observed at the distal phalanx base of the little finger suggests regional variability in local bone quality at anatomically comparable sites, with potential relevance to avulsion-type injuries and fixation planning at this level [14,15].

In addition to the descriptively observed inter-digital variation, the observed intra-digital variability in BMD distribution may also have clinically relevant implications. Differences in bone density between phalangeal levels within the same digit may correspond to variations in local mechanical loading at tendon insertion sites. For instance, the insertion of the flexor digitorum profundus at the distal phalanx and the flexor digitorum superficialis at the middle phalanx are subject to distinct biomechanical demands, which may be reflected in the local bone quality. Experimental studies have demonstrated that bone quality influences the mechanical performance of tendon-to-bone fixation using anchors, with lower bone density associated with reduced fixation strength [8,9,10,16]. These variations may have implications for fixation strategies, as the mechanical environment and bone stock at each insertion site may influence surgical approaches or implant selection. Therefore, intra-digital BMD mapping may provide additional information relevant to treatment strategies for tendon avulsions and reinsertion procedures by accounting for site-specific variations in bone quality.

From a fracture management perspective, the observed BMD distribution patterns may also have implications for fixation at the phalangeal bases, which are common sites for intra-articular and periarticular fractures. Variations in local bone quality at these levels may influence screw purchase, stability of fixation constructs, and the risk of fixation failure, particularly in small fragments or osteoporotic bone. Although the present study focused specifically on phalangeal bases and does not address the entire phalanx, the identification of site-specific variation in BMD suggests that fixation strategies in fractures involving these regions may benefit from consideration of local bone density [5,12,13,15,17].

These observations highlight the potential role of CT-derived BMD mapping as a source of additional information relevant to implant selection and fixation planning. Such information may be used in preoperative planning by identifying regions that may provide more favorable local bone support for fixation [16,17]. However, the extent to which such imaging-derived BMD metrics influence surgical decision-making and translate into improved clinical outcomes remains to be established. Prospective studies linking site-specific density measures to intraoperative fixation performance and postoperative outcomes are warranted to establish clinically relevant thresholds for secure fixation and to determine the practical value of incorporating BMD mapping into routine preoperative planning [15,17].

### Limitations

This study has some limitations. Only limited demographic information (age and sex) was available for the cadaveric specimens, while data on occupation, hand dominance, and comorbidities were not accessible. However, the primary aim of the study was not to investigate associations between bone mineral density (BMD) and demographic factors—an area that has been previously explored—but to perform a comparative assessment of BMD distribution between and within digits. Therefore, the lack of detailed demographic data is unlikely to have significantly affected the main findings.

The sample size was limited, as is common in cadaveric imaging studies, and therefore the findings should be interpreted primarily in a descriptive and exploratory context rather than as population-based normative reference values.

In addition, although the study incorporated repeated-measures statistical comparisons, it did not include mechanical validation or clinical outcome correlation. Although phantom-calibrated CT provides quantitative volumetric BMD values, the relationship between measured density at specific phalangeal bases and actual fixation strength was not directly evaluated, and therefore the findings cannot be directly extrapolated to all fracture types or fixation scenarios [4].

In addition, all ROI measurements were performed by a single observer, and formal intraobserver or interobserver reliability analyses were not performed. However, each final BMD value represented the mean of multiple ROI measurements obtained across consecutive axial slices within predefined three-dimensional anatomical boundaries. Standardized anatomical landmarks, multiplanar reconstructions, predefined anatomical boundaries, and three-dimensional projection guidance were consistently applied to minimize measurement variability throughout the analysis.

Future investigations should integrate HU-derived or phantom-calibrated BMD measurements with biomechanical pull-out testing and prospective clinical outcome data to help establish clinically relevant thresholds for secure fixation across digits and phalangeal levels [15,16]. Such studies would further clarify the translational value of site-specific density mapping in surgical decision-making.

## 5. Conclusions

Phantom-calibrated CT analysis demonstrated substantial intra-digital variability and descriptive inter-digital differences in bone mineral density (BMD) at the bases of the phalanges. The highest mean BMD values were observed at the proximal bases of the thumb and index finger, followed by the middle phalanx bases of the middle and ring fingers and the distal phalanx base of the little finger. Within digits, BMD distribution demonstrated level-specific patterns, with the highest mean BMD values occurring proximally in the thumb and index fingers, at the middle phalanx in the middle and ring fingers, and distally in the little finger. Repeated-measures ANOVA demonstrated statistically significant intra-digital differences in the thumb and index fingers, whereas no statistically significant inter-digital differences were identified across corresponding phalangeal levels.

Site-specific BMD mapping derived from CT may assist in preoperative planning by identifying regions of comparatively higher local bone density that may be relevant for anchor or screw placement. Further studies incorporating biomechanical validation and clinical outcome data are required to determine whether quantitative BMD assessment translates into improved fixation stability and surgical outcomes.

## Figures and Tables

**Table 1:** Descriptive inter- and intra-digital comparisons of bone mineral density (BMD).

Phalanx	Digit	Mean BMD (mg/cm^3^)	SD	*n*	Inter-Digital BMD Rank
Proximal	Thumb	276.35	91.36	10	1
Proximal	Index	270.52	78.44	10	2
Proximal	Middle	253.64	103.21	10	3
Proximal	Ring	249.18	52.22	9	4
Proximal	Little	230.65	62.63	9	5
Middle	Middle	266.41	101.01	10	1
Middle	Ring	263.89	69.30	9	2
Middle	Little	251.59	70.50	9	3
Middle	Index	244.80	98.21	10	4
Distal	Little	254.45	84.46	9	1
Distal	Ring	240.71	81.62	9	2
Distal	Middle	235.36	85.35	10	3
Distal	Thumb	212.58	45.70	10	4
Distal	Index	198.39	116.12	10	5

Abbreviations: BMD, bone mineral density; SD, standard deviation.

**Table 2:** Inter-digital repeated-measures ANOVA comparisons of BMD across corresponding phalangeal levels.

Phalangeal Level	F	*p*-Value
Proximal	1.99	0.120
Middle	0.712	0.555
Distal	1.27	0.304

Abbreviations: ANOVA, analysis of variance; F, F-statistic; *p*-value, probability value.

**Table 3:** Intra-digital repeated-measures ANOVA comparisons of BMD across phalangeal levels.

Digit	Comparison	F	*p*-Value
Thumb	Proximal vs. Distal	8.39	0.018
Index	Proximal vs. Middle vs. Distal	6.3	0.008
Middle	Proximal vs. Middle vs. Distal	0.672	0.532
Ring	Proximal vs. Middle vs. Distal	0.926	0.416
Little	Proximal vs. Middle vs. Distal	1.42	0.271

Abbreviations: ANOVA, analysis of variance; F, F-statistic; *p*-value, probability value.

## Data Availability

The datasets generated and analyzed during the current study (including HU measurements and phantom-calibrated BMD values) are available from the corresponding author upon reasonable request. The data are not publicly archived due to institutional restrictions related to cadaveric specimen use.

## Supplementary Materials

The following supporting information can be downloaded at: https://www.mdpi.com/article/10.3390/diagnostics16121843/s1, Tables S1–S10, presenting detailed minimum, maximum, and mean BMD measurements per specimen and phalanx. Table S1: H-1 BMD Measurements; Table S2: H-2 BMD Measurements; Table S3: H-3 BMD Measurements; Table S4: H-4 BMD Measurements; Table S5: H-5 BMD Measurements; Table S6: H-6 BMD Measurements; Table S7: H-7 BMD Measurements; Table S8: H-8 BMD Measurements; Table S9: H-9 BMD Measurements; Table S10: H-10 BMD Measurements.

## Abbreviations

The following abbreviations are used in this manuscript:

BMD	Bone mineral density
CT	Computed tomography
HU	Hounsfield units
ROI	Region of interest
MPR	Multiplanar reconstruction
PIP	Proximal interphalangeal
ANOVA	Analysis of variance
